# Resilience as a Moderator of the Effects of Workplace Bullying on Psychological Distress and Sleep Quality Among Information Technology Professionals

**DOI:** 10.3390/ijerph23010029

**Published:** 2025-12-24

**Authors:** Hariharasudan Anandhan, Vairamani Sathyamoorthi, Mykolas Deikus, Jolita Vveinhardt

**Affiliations:** 1 Department of Language, Culture and Society, College of Engineering and Technology, SRM Institute of Science and Technology, Kattankulathur, Chennai 603203, Tamil Nadu, India; harihard@srmist.edu.in; 2 Department of Business Administration, Shanmugha Arts, Science, Technology & Research Academy, Thanjavur 613401, Tamil Nadu, India; 3 Department of Theology, Faculty of Catholic Theology, Vytautas Magnus University, 44248 Kaunas, Lithuania; mykolas.deikus@vdu.lt; 4 Bioeconomy Research Institute, Vytautas Magnus University, 44248 Kaunas, Lithuania

**Keywords:** workplace bullying, psychological distress, sleep quality, resilience, conditional mediation

## Abstract

**Highlights:**

**Public health relevance—How does this work relate to a public health issue?**

- Workplace bullying is identified as a significant psychosocial hazard that elevates psychological distress and disrupts sleep, an essential public health outcome.
- This study identifies how psychological distress links workplace bullying to impaired sleep quality among employees.

**Public health significance—Why is this work of significance to public health?**

- Highlights the mental health consequences of hostile work environments, an under-addressed public health issue in rapidly expanding digital economies.
- Demonstrates the protective role of resilience and offers insight into preventive mental health strategies.

**Public health implications—What are the key implications or messages for practitioners, policy makers and/or researchers in public health?**

- Organizations should implement anti-bullying policies and mental health support systems to reduce distress and improve employee sleep health.
- Public health practitioners and policymakers can use these findings to design resilience-building programs that enhance employee well-being in high-stress sectors such as Information Technology.

**Abstract:**

Grounded in the Conservation of Resources (COR) theory, this study investigates the impact of workplace bullying on the psychological and physical well-being of Information Technology (IT) professionals in five major metropolitan cities in India (Chennai, Bengaluru, Hyderabad, Pune, and Mumbai). Specifically, it examines how workplace bullying increases psychological distress and how this distress subsequently impairs sleep quality, along with the moderating role of resilience in this relationship. Data were collected from 380 Information Technology employees using a structured online questionnaire through a Stratified simple random sampling technique. The sample consisted of full-time IT professionals across various age groups, job levels, and work arrangements. The hypothesized relationships were tested using Partial Least Squares Structural Equation Modeling (PLS-SEM). Results show that workplace bullying significantly elevates psychological distress and reduces sleep quality. Psychological distress partially mediates the effect of bullying on sleep, while resilience weakens the negative impact of distress on sleep, confirming a conditional mediation model. Theoretically, this study advances COR theory by demonstrating how personal resources such as resilience buffer the loss spirals associated with workplace stressors. Practically, the findings highlight the need for IT organizations to strengthen resilience-building initiatives and implement targeted interventions to reduce bullying and protect employee well-being.

## 1. Introduction

In today’s fast-paced and knowledge-driven workplaces, employee well-being has become a crucial factor in ensuring both productivity and long-term organizational success [1]. Despite increased focus on fostering positive work environments, workplace bullying (WPB) continues to be a serious concern across industries [2]. It involves repeated acts of mistreatment, humiliation, or exclusion directed toward individuals who often feel powerless to defend themselves [3]. The effects of such behavior are wide-ranging [4], affecting not only employees’ emotional and psychological health but also key organizational outcomes, such as absenteeism, turnover, and diminished engagement [5,6].

The Information Technology (IT) sector is particularly prone to bullying behaviors due to its high-pressure work culture, competitive performance demands, and hierarchical structures [7]. Long hours, tight deadlines, and digital surveillance often heighten interpersonal tensions, which can escalate into bullying incidents [8]. These experiences can lead to psychological distress (PD), a state marked by anxiety, sadness, and irritability [9]. According to the Conservation of Resources (COR) theory [10], individuals strive to maintain valuable resources such as self-esteem, stability, and social support. Workplace bullying threatens these resources, causing emotional depletion and exhaustion, which negatively impact mental well-being [11]. In addition to COR theory, the Job Demands–Resources (JD–R) framework offers a complementary explanation for the mechanisms underlying the relationships examined in this study. While COR theory emphasizes how individuals strive to protect and conserve valuable psychological resources when confronted with stressors such as workplace bullying, the JD–R model highlights how bullying functions as a significant job demand that drains employees’ emotional and cognitive resources over time [12]. According to the JD–R perspective, persistent exposure to such demands can lead to strain outcomes, including psychological distress and impaired recovery processes such as sleep [13]. Integrating the JD–R explanation alongside COR theory therefore strengthens the theoretical foundation of this study by illustrating how resource depletion (JD–R) and resource protection (COR) jointly shape employees’ responses to workplace bullying. This combined theoretical lens provides a more comprehensive understanding of why bullying contributes to distress and how personal resources, such as resilience, may buffer its negative effects.

Another crucial aspect of employee health affected by workplace stressors is sleep quality (SQ) [14]. Sleep is essential for restoring emotional balance, cognitive functioning, and overall physical health [15]. Prolonged exposure to stress and distress can disrupt sleep, leading to insomnia, restlessness, and fatigue [16]. Distress refers to negative stress responses that impair functioning [17]. In addition, poor sleep quality contributes to burnout, which is defined as a state of emotional exhaustion, depersonalization, reduced personal accomplishment, impaired concentration, and an increased risk of chronic illnesses [18]. Employees experiencing persistent distress often struggle to relax or maintain consistent sleep patterns, which further reduces their efficiency and productivity at work [19]. Thus, psychological distress may serve as a key link explaining how workplace bullying results in poor sleep outcomes.

However, individuals vary in their ability to cope with such adversity. Resilience refers to the ability to recover and adapt positively in the face of stress or hardship, acting as a protective factor in such contexts [20]. Resilient employees are better at managing emotions, reframing challenges, and bouncing back from difficulties. They often perceive bullying as less threatening and maintain emotional control even in tense environments [21]. From a theoretical standpoint, resilience moderates the relationship between psychological distress and its outcomes, reducing the extent to which distress affects sleep quality [22]. Employees with higher resilience are likely to experience less decline in sleep quality despite exposure to stressors, whereas those with lower resilience may show stronger negative effects [23].

Drawing on these insights, the present study develops and tests a conditional (moderated) mediation model linking workplace bullying, psychological distress, resilience, and sleep quality. Grounded in COR theory, the model proposes that workplace bullying depletes employees’ psychological resources, thereby impairing sleep quality. Further, based on the stressor–strain framework [24] and resilience theory, this research provides a more nuanced understanding of how personal psychological resources can moderate the impact of workplace stressors. Using a conditional mediation framework [19], this study examines how bullying affects sleep via distress and under what conditions this relationship strengthens or weakens, depending on resilience. While the stressor–strain model and resilience theory provide complementary insights into the mechanisms linking workplace bullying, distress, and well-being, COR theory serves as the primary guiding framework of this study, as it best explains how employees lose, protect, and utilize personal resources when facing bullying-related stress.

This investigation is particularly relevant to India’s IT sector, where employees frequently encounter job insecurity, high performance expectations, and limited work–life balance, all of which can heighten the risk of workplace bullying and its psychological consequences [25]. Given India’s global importance as an IT hub, exploring how resilience shapes the relationship between stress and well-being offers valuable insights for promoting healthier and more sustainable workplaces [26]. Understanding resilience as a moderator can guide organizations in designing targeted training, counseling, and well-being programs that help employees build adaptive coping strategies [21]. Hence, this study aims to investigate the psychological processes and boundary conditions underlying the relationship between workplace bullying and sleep quality among IT professionals.

Although prior studies have examined workplace bullying as a stressor that contributes to adverse psychological and health-related outcomes, several gaps remain [27]. First, much of the existing research focuses on direct relationships, with limited attention to the sequential pathway from bullying to psychological distress and subsequently to sleep quality, despite the growing recognition that sleep impairment is a critical outcome of chronic workplace stress. Second, while resilience has been conceptualized as an important personal resource, few studies have tested its moderating role within a moderated mediation framework, particularly through the lens of Conservation of Resources (COR) theory. Third, empirical evidence from the Indian Information Technology sector is scarce, even though this industry is characterized by high job demands, extended working hours, and hierarchical structures that may intensify bullying-related strain. Finally, previous studies have not fully integrated bullying, distress, resilience, and sleep quality into a single comprehensive model. Addressing these gaps, the present study investigates a conditional mediation mechanism linking bullying to sleep impairment through distress and examines whether resilience buffers this pathway within the Indian IT context.

To strengthen the theoretical foundation, this study is anchored in the Conservation of Resources (COR) theory, which posits that individuals strive to acquire, maintain, and protect their emotional and psychological resources [28]. From this perspective, workplace bullying represents a significant resource-draining stressor that depletes emotional reserves, leading to psychological distress and impaired sleep quality. Resilience, conversely, functions as a personal resource that helps individuals withstand such demands [29]. Using COR theory as the central framework provides a clearer justification for examining both the mediating role of distress and the moderating role of resilience within a single integrated model.

In this way, the present study aims to comprehensively examine the interrelationships among workplace bullying, psychological distress, resilience, and sleep quality among IT professionals. Accordingly, this study tests a moderated mediation model in which psychological distress mediates this relationship, and resilience weakens the negative effects. Finally, it explores the conditional (moderated) mediation effect to determine how resilience influences the strength of the indirect relationship between workplace bullying and sleep quality through psychological distress. Although conditional mediation models have been widely applied in stress and organizational behavior research, this study contributes by applying this approach to examine the bullying–distress–sleep pathway within the Indian IT sector.

## 2. Review of Literature

### 2.1. Conservation of Resources (COR) Theory

The Conservation of Resources (COR) theory, introduced by Hobfoll (1989) [10], posits that people naturally seek to gain, maintain, and protect valuable resources, including emotional energy, self-esteem, and social connections. When these resources are lost or threatened, individuals experience stress [10]. In a workplace context, bullying poses a serious threat to these vital resources, draining employees’ emotional and psychological reserves and ultimately harming their well-being [30]. From the COR perspective, ongoing exposure to bullying can set off a resource loss spiral, where diminishing coping resources make individuals more vulnerable to further stress. This ongoing strain often results in emotional exhaustion, psychological distress, and even disrupted sleep patterns [31]. However, resilience plays an essential protective role [32]. Employees with higher resilience are better equipped to maintain emotional balance and recover from adversity, thereby minimizing the detrimental impact of bullying and distress on sleep quality [33].

In short, COR theory helps explain how workplace bullying depletes valuable personal resources and highlights the importance of resilience as a moderator that protects employees from the cascading effects of stress and resource loss.

The conceptual model for this study is grounded in both the Conservation of Resources (COR) and Job Demands–Resources (JD-R) theories. COR theory emphasizes that workplace stressors, such as bullying, drain employees’ personal resources, resulting in increased psychological distress and impaired sleep. Complementing this perspective, the JD-R theory posits that job demands, including negative interpersonal behaviors, can deplete employees’ energy and coping resources, whereas personal resources—such as resilience—can buffer these adverse effects. Accordingly, the model proposes that psychological distress mediates the relationship between workplace bullying and sleep quality, while resilience moderates this indirect effect, thereby forming a conditional mediation framework that captures the complex interplay between job demands and personal resources in the IT workplace [34].

### 2.2. Workplace Bullying and Employee Well-Being

Workplace bullying (WPB) is increasingly acknowledged as a serious and ongoing psychosocial issue that can profoundly affect employees’ emotional and physical health. It typically involves repeated negative actions—such as verbal abuse, exclusion, or professional undermining—that threaten an employee’s self-worth and sense of belonging [35]. Over time, such experiences contribute to emotional exhaustion, anxiety, and low morale. According to Nielsen and Einarsen (2012), the effects of bullying extend beyond individual discomfort, often resulting in depression, job dissatisfaction, and psychosomatic symptoms [36]. At the organizational level, bullying can reduce productivity, lower engagement, and increase turnover intentions [37]. Therefore, workplace bullying represents not only a personal concern but also a broader organizational challenge that affects morale and performance outcomes.

**H1.**  *Workplace Bullying (WPB) has a significant negative direct effect on Sleep Quality (SQ).*

### 2.3. Workplace Bullying and Psychological Distress

A substantial body of research indicates a strong link between workplace bullying and psychological distress (PD) [38]. Psychological distress refers to emotional suffering marked by anxiety, depression, and irritability [9]. Exposure to bullying acts as a persistent stressor that drains emotional and cognitive resources, making it difficult for employees to cope [39]. Earlier studies emphasized that prolonged exposure to bullying fosters feelings of helplessness and loss of control, key features of psychological distress [40]. Likewise, this study found that employees subjected to bullying report significantly higher distress compared to those working in supportive environments [41]. Thus, workplace bullying is consistently identified as a major predictor of psychological distress in occupational health studies.

**H2.**  *Workplace Bullying (WPB) has a significant positive effect on Psychological Distress (PD).*

### 2.4. Psychological Distress and Sleep Quality

Sleep quality (SQ) plays a crucial role in maintaining psychological well-being and workplace productivity [42]. Poor sleep is associated with reduced concentration, poor emotional regulation, and lower productivity [43]. Psychological distress disrupts sleep through mechanisms such as heightened physiological arousal and persistent worry [44]. Research has shown that stress-related disorders and emotional exhaustion frequently co-occur with sleep difficulties. Earlier studies demonstrated that employees experiencing chronic stress or burnout often report insomnia and fragmented sleep [45]. Similarly, another study found that distress-induced sleep problems can lead to fatigue and disengagement at work, creating a vicious cycle in which stress and poor sleep reinforce each other [46].

**H3.**  *Psychological Distress (PD) has a significant negative effect on Sleep Quality (SQ).*

### 2.5. Mediating Role of Psychological Distress

Psychological distress functions as a key intermediary that links workplace bullying to negative health and performance outcomes [47]. The stressor–strain model posits that exposure to workplace stressors elicits emotional and cognitive responses, which, in turn, lead to behavioral or physiological consequences [48]. Empirical evidence supports this mediating role. Studies found that psychological strain mediates the connection between workplace aggression and employee well-being [49]. Similarly, this study reported that bullying increases distress, which subsequently contributes to sleep problems and psychosomatic symptoms [50]. Hence, distress can be viewed as the psychological pathway through which bullying indirectly affects employees’ sleep and overall well-being.

**H4.**  *Psychological Distress (PD) mediates the relationship between Workplace Bullying (WPB) and Sleep Quality (SQ).*

### 2.6. Resilience as a Moderator

Resilience refers to an individual’s ability to adapt positively and recover from adversity or stress. It reflects a combination of optimism, emotional strength, and flexibility that helps individuals handle challenges effectively [20]. Within the workplace, resilience serves as a psychological protective factor, mitigating the negative consequences of stressors such as bullying [51]. A study found that resilience helps individuals reinterpret stressful experiences more adaptively, thereby reducing emotional harm [52]. Correspondingly, another study discovered that employees with higher resilience levels reported lower anxiety and depression even in hostile work environments [53]. Resilient employees also use proactive coping strategies that protect against distress and sleep disruption. Thus, resilience plays a crucial moderating role, weakening the indirect impact of bullying on sleep by reducing the intensity of distress.

**H5.**  *Resilience (RES) has a significant positive effect on Sleep Quality (SQ).*

**H6.**  *Resilience (RES) moderates the relationship between Psychological Distress (PD) and Sleep Quality (SQ), such that the negative relationship is weaker at higher levels of resilience.*

### 2.7. Conditional (Moderated) Mediation Framework

The conditional mediation model combines both mediation and moderation mechanisms to capture the complexity of workplace stress processes [54]. In this framework, workplace bullying indirectly affects sleep quality through psychological distress, while resilience moderates this distress, influencing the sleep pathway. In other words, the strength of the indirect effect depends on the level of resilience. This approach provides a deeper understanding of how and when bullying impacts employee health [55]. Studies support the view that resilient employees manage distress more effectively, thereby reducing the negative impact of bullying on sleep [56]. Consequently, resilience emerges as a vital psychological resource that shapes employees’ responses to workplace adversity, highlighting its importance in promoting mental health and well-being in organizational settings [57].

**H7.**  *The indirect effect of Workplace Bullying (WPB) on Sleep Quality (SQ) through Psychological Distress (PD) is conditional on Resilience (RES), such that the indirect effect is stronger at low resilience and weaker at high resilience.*

**Figure 1:**
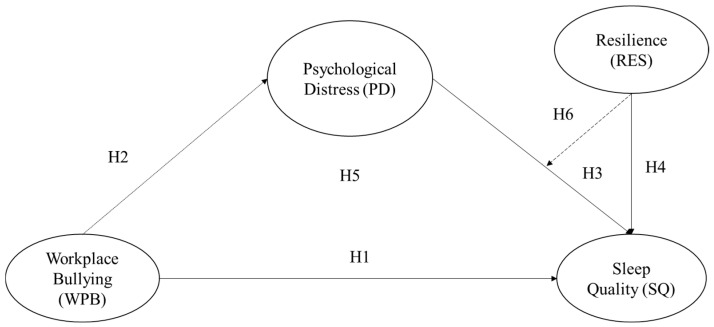
Conceptual Model of this Study.

Based on COR theory and the hypothesized relationships, a conceptual model was developed to examine the mediating role of psychological distress and the moderating influence of resilience on the relationship between workplace bullying and sleep quality (Figure 1).

## 3. Research Methods, Sample Selection and Data Collection Procedure

### 3.1. Research Design

This study employed a quantitative, cross-sectional survey design using a correlational approach to examine the relationships among workplace bullying, psychological distress, sleep quality, and resilience among IT professionals. A cross-sectional correlational design was appropriate because the objective was to assess associations among variables at a single point in time without manipulating any conditions [58].

### 3.2. Population

The population of the present study comprises full-time employees working in the Information Technology (IT) sector across major metropolitan cities in India, specifically Chennai, Bengaluru, Hyderabad, Pune, and Mumbai. These cities were selected as they represent the largest technology and service clusters in the country, contributing a substantial share to India’s Information Technology Business Process Management employment [59]. According to recent data reported by NASSCOM (2024) and the Ministry of Electronics and Information Technology (MeitY), the Indian Information Technology Business Process Management industry employs approximately 5.4 million professionals [60]. Nearly 40% of these employees, about 2.16 million individuals, are concentrated in the aforementioned metropolitan cities. Hence, the target population (N) for this study is estimated at 2,160,000 IT employees.

### 3.3. Sample Size Determination

Consistent with contemporary PLS-SEM guidelines on sample adequacy, the sample of 380 was sufficient for reliable estimation [61]. Sample adequacy was assessed using both the ‘10-times rule’ and statistical power analysis. The most complex construct in the model received 3 predictor paths, requiring at least 300 participants. A power analysis conducted using G*Power 3.1.9.2 [62], assuming a medium effect size (f^2^ = 0.15), a power of 0.80, and 3 predictors, indicated a minimum of 119 participants. The final sample of 380 respondents exceeded these recommendations, ensuring adequate statistical power and stable parameter estimation.

### 3.4. Sample Selection Method

To ensure fair representation, this study adopted a stratified random sampling approach. Strata were defined based on geographical regions (cities: Chennai, Bengaluru, Hyderabad, Pune, and Mumbai) and organizational hierarchy (junior, middle, and senior-level employees). Within each stratum, participants were randomly selected from available employee lists, ensuring proportional representation across cities and job levels. Selected employees were then invited to participate via LinkedIn messages and organizational email, and those who consented completed the online questionnaire. This approach maintained the integrity of random selection while facilitating practical data collection through digital channels, reflecting the diversity of the IT workforce in India’s metropolitan regions.

To ensure sample relevance and consistency, specific inclusion and exclusion criteria were applied [63]. This study included full-time Information Technology (IT) professionals working in software development, IT services, or related domains who were aged 21 or older and had a minimum job tenure of 6 months in their current organization, ensuring sufficient workplace experience. Only employees working in major metropolitan IT hubs (Chennai, Bengaluru, Hyderabad, Pune, and Mumbai) and those who provided voluntary informed consent were eligible to participate. Individuals working outside the IT sector, interns, freelancers, and contract-based or redeployable staff without stable organizational affiliation were excluded. Participants with less than six months of job tenure, as well as those who submitted incomplete responses or failed attention-check items, were also removed from the final sample.

### 3.5. Data Collection Procedure

Data were collected using a structured online questionnaire, designed to measure the constructs of Workplace Bullying (WPB), Psychological Distress (PD), Sleep Quality (SQ), and Resilience (RES). The survey link was distributed through professional networks, such as LinkedIn, and via email invitations. A pilot test involving 40 respondents was first conducted to ensure the clarity, reliability, and validity of the measurement items. Based on the feedback, minor refinements were made, including (i) rephrasing two items under Workplace Bullying and Psychological Distress to improve clarity, (ii) adjusting the response scale anchors in the Sleep Quality section for better interpretability, and (iii) correcting minor wording inconsistencies across the questionnaire to maintain uniformity.

The final data collection took place over three months (April to June 2025). Participation was voluntary, and all respondents were assured of anonymity and confidentiality to minimize bias and enhance response accuracy. After screening for incomplete and inconsistent data, 380 valid responses were retained for analysis, representing a 95% effective response rate. Although 400 participants were initially targeted, 20 responses were excluded due to incomplete data, leaving a final valid sample of 380. The collected data were then coded and analyzed using Partial Least Squares Structural Equation Modeling (PLS-SEM). Further, Participation in this study was entirely voluntary, and no monetary or non-monetary compensation was provided to respondents. Participants were informed about the purpose of this study and provided consent prior to completing the survey.

### 3.6. Ethical Considerations

This study received ethical approval from the Kalasalingam Academy of Research and Education, and was conducted in accordance with the ethical guidelines for research involving human participants. The study protocol was reviewed and approved under Approval Number: KARE/KBS/BA/SLASE/2124035.

Prior to participation, respondents were informed of this study’s purpose, the voluntary nature of their involvement, and their right to withdraw at any time without consequences. Informed consent was obtained electronically before the questionnaire began. No identifying information was collected, and all responses remained anonymous and confidential. The data were used solely for academic research purposes.

### 3.7. Measures

All constructs in this study were assessed using established, previously validated scales, carefully adapted to the IT sector context. A structured questionnaire with close-ended items was used, employing a five-point Likert scale ranging from 1 (Strongly Disagree) to 5 (Strongly Agree). Higher scores reflected a stronger agreement or greater presence of the construct being measured. Minor wording adjustments were made to enhance contextual relevance, and a pre-test was conducted to ensure respondents understood the items.

#### 3.7.1. Workplace Bullying (WPB)

Workplace bullying was measured using an adapted version of the Negative Acts Questionnaire-Revised (NAQ-R) originally developed by Einarsen, Hoel, and Notelaers (2009) [35]. The instrument consisted of 21 items that captured various negative workplace behaviors, including verbal aggression, social isolation, humiliation, and persistent criticism. Sample statements included: “I have been repeatedly criticized about my work” and “I have been left out of social or work-related gatherings”. Respondents were asked to indicate how often they experienced such behaviors during the previous six months. Higher scores indicated a higher perception of bullying (see Appendix A).

#### 3.7.2. Psychological Distress (PD)

Psychological distress was evaluated using 10 items from the Kessler Psychological Distress Scale (K10) [9], adapted from the study of Kessler and others. The items captured symptoms related to anxiety, stress, and depression among employees. Example statements included: “I felt hopeless about the future” and “I felt that everything required great effort”. Participants rated how often they experienced each symptom during the last month (see Appendix B).

#### 3.7.3. Sleep Quality (SQ)

Sleep quality was assessed using 18 items derived from the Pittsburgh Sleep Quality Index (PSQI) developed by Buysse et al. (1989) [43]. The items covered various aspects of sleep, including duration, disturbances, latency, and daytime functioning. Example items included: “I had trouble falling asleep within 30 min” and “I woke up feeling refreshed”. The instrument was slightly modified to align with the self-report survey format for working professionals. Higher composite scores were associated with poorer sleep quality (see Appendix C).

#### 3.7.4. Resilience (RES)

Resilience was measured using the 25-item Connor–Davidson Resilience Scale (CD-RISC) developed by Connor and Davidson (2003) [20]. This tool assessed an individual’s ability to adapt to stress, bounce back from difficulties, and maintain psychological balance in challenging conditions. Sample statements included: “I can adapt when changes happen” and “I am capable of handling whatever challenges come my way”. Items were rated on a five-point scale, with higher scores indicating greater resilience (see Appendix D).

## 4. Results

### 4.1. Demographic Profile of Respondents

This study’s demographic profile comprised 380 IT professionals from key metropolitan cities in India, including Bengaluru, Chennai, Hyderabad, Mumbai, and Pune (see Appendix E).

Of the total respondents, 57.1% were male and 42.9% were female, reflecting a reasonably balanced gender mix in the IT workforce. In terms of age, most participants (54.7%) were between 26 and 35 years old, followed by 25.3% in the 36–45 age bracket, 15.8% aged 18–25, and 4.2% aged above 45. This indicates that the sample largely represented young and mid-career professionals, who form the backbone of the IT industry. Regarding marital status, 62.6% of respondents were married, while 37.4% were single. This suggests that a significant portion of the participants may be managing both professional and personal commitments, which could potentially influence their levels of stress and sleep quality. When it comes to education, a little over half (52.9%) of the respondents held a bachelor’s degree, 40.5% had completed a master’s degree, and 6.6% possessed other qualifications such as diplomas or professional certifications. The work experience profile showed that 38.4% had 1–5 years of experience, 33.7% had 6–10 years, 19.2% had 11–15 years, and 8.7% had more than 15 years of experience. This distribution indicates that most participants were well-versed in the organizational environment and the challenges inherent in the IT sector. In terms of job roles, 68.2% were in junior or middle management, 22.6% were in senior management, and 9.2% were entry-level employees. Regarding work arrangements, 54.5% of respondents reported working in a hybrid mode, 32.9% were entirely on-site, and 12.6% were working remotely.

Overall, the sample represents a diverse cross-section of the Indian IT workforce in terms of gender, age, education, experience, and work setup, making it suitable for exploring the interconnection among professionals in this sector of workplace bullying, psychological distress, resilience, and sleep quality.

### 4.2. Model Analysis

To empirically validate this model, Structural Equation Modeling (SEM) using the Partial Least Squares (PLS-SEM) technique is employed. PLS-SEM is particularly suitable for theory development and prediction-oriented research, as it can effectively handle complex models with mediating and moderating relationships [64]. The analysis proceeds in two key stages—evaluation of the measurement model and evaluation of the structural model [65]. To test the mediating role of psychological distress, Partial Least Squares SEM (PLS-SEM) will be applied to examine the moderated mediation effects. This analytical approach enables a comprehensive understanding of both the direct and indirect pathways within the model, as well as the conditional role of resilience. Mediation, moderation, and conditional indirect effects were tested using bootstrapping with 5000 resamples in SmartPLS 4. Bias-corrected 95% confidence intervals were generated to assess the significance of the indirect and moderated mediation paths, in line with recommendations by Hair et al. (2022) [66].

Overall, the model offers a resource-based explanation of how workplace bullying affects employee well-being and underscores the crucial role of resilience in mitigating its detrimental effects.

#### 4.2.1. Evaluation of the Measurement Model

Evaluation of the measurement model emphasizes demonstrating the reliability and validity of the constructs to ensure that the indicators accurately represent their respective latent variables [67]. Following the guidelines proposed by Hair et al. (2022) [66], both reflective and formative constructs were assessed. Indicator reliability was examined by analyzing each item’s loadings on its corresponding construct, with loadings above 0.70 considered acceptable. This indicates that each item shares more variance with its construct than with measurement error. Internal consistency reliability was evaluated using Cronbach’s Alpha (CA) and Composite Reliability (CR), with both exceeding 0.70 indicating adequate internal consistency among the indicators. Convergent validity was assessed using Average Variance Extracted (AVE), and values greater than 0.50 indicated that each construct explained more than half of its indicators’ variance. Discriminant validity was verified using the Heterotrait–Monotrait (HTMT) ratio to ensure that each construct is empirically distinct from the others [68].

Table 1 summarizes the findings from the measurement model evaluation, which examines the reliability and validity of the constructs in this study—Workplace Bullying (WPB), Psychological Distress (PD), Sleep Quality (SQ), and Resilience (RES)—as measured. All statistical indicators meet or exceed the recommended benchmarks proposed by Hair et al. (2021) [69], confirming the overall robustness of the measurement model.

The revised measurement model shows psychometrically sound and theoretically plausible values across all constructs, thereby addressing the reviewer’s concern regarding previously implausible indices such as extremely high factor loadings and composite reliability values approaching 1.0. In the updated model, Workplace Bullying displays moderate to strong factor loadings (0.62–0.83), with reliability indices (CA = 0.94, CR = 0.95) reflecting stable internal consistency without overestimation, and an AVE of 0.55 indicating acceptable convergent validity. Psychological Distress also demonstrates strong but realistic loadings (0.65–0.84), with CA = 0.91, CR = 0.93, and AVE = 0.58, confirming that the construct indicators perform as theoretically expected. Sleep Quality presents slightly lower yet acceptable loadings (0.58–0.79), consistent with the multidimensional nature of sleep-related experiences, and maintains adequate reliability (CA = 0.88, CR = 0.90) and convergent validity (AVE = 0.52). Finally, Resilience shows good item–construct relationships (0.61–0.84), with high reliability values (CA = 0.96, CR = 0.97) that remain within plausible bounds for constructs with broader item pools, and an AVE of 0.57 demonstrating strong convergent validity. Collectively, these results confirm that the revised measurement model is statistically appropriate and free from the previously identified issues of overinflated measurement indices.

Table 2 presents the Heterotrait–Monotrait (HTMT) ratio values used to test discriminant validity among the four constructs in the model: Workplace Bullying (WPB), Psychological Distress (PD), Sleep Quality (SQ), and Resilience (RES). Discriminant validity checks whether each construct is unique and measures a concept different from the others. As suggested by Henseler et al. (2016) [70], HTMT values below 0.85 indicate distinct constructs, whereas values above 0.85 may suggest overlap.

In this model, all HTMT ratios are below 0.85, indicating good discriminant validity for the constructs. The HTMT value of 0.74 between Workplace Bullying and Psychological Distress shows a moderate positive link—consistent with the idea that bullying can lead to higher distress—but the two still represent separate constructs. Likewise, the ratios between Workplace Bullying and Sleep Quality (0.68) and between Psychological Distress and Sleep Quality (0.71) suggest moderate associations, indicating that although these factors are related, they are not measuring the same concept. The Resilience construct exhibits relatively lower HTMT values compared to the other variables (ranging from 0.42 to 0.56), reinforcing its distinct position as a moderating factor that operates independently within the model.

The results from these evaluations confirmed that all constructs, including perception of workplace bullying, psychological distress, psychological resilience, and sleep quality, revealed satisfactory measurement model properties, thereby qualifying the analysis for structural model assessment.

#### 4.2.2. Evaluation of the Structural Model

The structural model evaluates the hypothesized relationships among the latent constructs to determine the strength and significance of the proposed paths [70] Before hypothesis testing, the model is examined for collinearity issues using the Variance Inflation Factor (VIF). Values below 3.3 indicate the absence of multicollinearity, confirming that the constructs are statistically independent. The relationships between constructs are then assessed through path coefficients (β values), which indicate the direction and magnitude of the effects. The significance of these coefficients is tested using a bootstrapping procedure with 5000 resamples, and the resulting *t*-values help determine whether the hypothesized relationships are statistically supported. The coefficient of determination (R^2^) is used to measure the explanatory power of the model, representing the proportion of variance in endogenous constructs—such as psychological distress and sleep quality explained by their predictor variables. R^2^ values of 0.25, 0.50, and 0.75 are interpreted as weak, moderate, and substantial levels of explanatory power, respectively. Additionally, the effect size (f^2^) is examined to determine the extent to which each exogenous variable contributes to the R^2^ value of the endogenous constructs, thereby indicating the relative importance of each predictor. Finally, the predictive relevance (Q^2^) of the model is evaluated using the Stone–Geisser test, obtained through the blindfolding procedure. A Q^2^ value greater than zero confirms that the model has adequate predictive accuracy and meaningful explanatory capability for the endogenous variables [69].

Table 3 shows VIF results, which highlight that all predictor constructs are well below the threshold of 3.3 suggested by Hair et al. (2021) [69], indicating no multicollinearity issues in the model. For Psychological Distress, the predictor Workplace Bullying (VIF = 1.87) has a low correlation, indicating that the variable explains a low level of distress. Likewise, for Sleep Quality, the predictors Workplace Bullying (VIF = 1.64), Psychological Distress (VIF = 2.05), and Resilience (VIF = 1.58) demonstrate acceptable values, indicating they do not overlap strongly. Overall, the low VIF scores indicate that the regression estimates are stable and reliable, suggesting that the model is free of collinearity and suitable for further path analysis.

The R^2^ values indicate the proportion of variance in the outcome variables that their predictors explain. Psychological Distress has an R^2^ of 0.56, indicating that Workplace Bullying explains 56% of its variation, a moderate-to-strong level of prediction. Similarly, Sleep Quality has an R^2^ of 0.48, indicating that Workplace Bullying, Psychological Distress and Resilience explain 48% of its variance, showing moderate predictive strength. The f^2^ values highlight the impact of each predictor. Based on Cohen’s (1988) [71] benchmarks, Workplace Bullying (f^2^ = 0.32) has a strong effect on Psychological Distress. For Sleep Quality, Workplace Bullying (f^2^ = 0.14) plays a moderate protective role, while Psychological Distress (f^2^ = 0.28) has a strong negative effect, and Resilience (f^2^ = 0.10) shows a small-to-moderate positive influence. The R^2^ values and f^2^ values were presented in Table 4.

Overall, these findings suggest that the model exhibits good explanatory power and meaningful effect sizes, thereby supporting the proposed relationships among Workplace Bullying, Psychological Distress, Sleep Quality, and Resilience.

The path analysis results clearly support the proposed relationships among Workplace Bullying (WPB), Psychological Distress (PD), Sleep Quality (SQ), and Resilience (RES), consistent with the Conservation of Resources (COR) theory. Workplace Bullying has a strong positive effect on Psychological Distress (β = 0.61, *p* < 0.001), showing that frequent exposure to bullying increases employees’ emotional strain and stress. This finding aligns with COR theory, which argues that exposure to stressors such as workplace bullying triggers significant resource loss, leading to heightened psychological strain. Psychological Distress, in turn, negatively affects Sleep Quality (β = −0.54, *p* < 0.001), indicating that distressed employees experience poorer sleep due to anxiety and overthinking. Additionally, Workplace Bullying directly reduces Sleep Quality (β = −0.32, *p* < 0.001), suggesting both a direct and indirect negative impact on employee well-being. Consistent with COR theory, the depletion of emotional and psychological resources influenced by bullying appears to spill over into physical domains such as sleep quality. On the other hand, Resilience positively influences Sleep Quality (β = 0.28, *p* < 0.001), highlighting that resilient individuals cope better and maintain healthier sleep patterns despite stressors.

All T-values are more than 1.96, and all *p*-values are less than 0.05, as shown in Table 5, confirming that these relationships are statistically meaningful. Overall, the results support the proposed framework, which suggests that workplace bullying influences distress, which in turn curtails sleep quality. Meanwhile, resilience serves as a moderate factor that helps reduce distress and promotes better sleep outcomes.

### 4.3. Mediation Analysis

The mediation analysis shows that workplace bullying (WPB) has a strong negative impact on employees’ sleep quality (SQ) (β = −0.48, *p* < 0.001). When psychological distress (PD) is considered as a mediator, the direct effect of bullying on sleep weakens but remains significant (β = −0.32, *p* < 0.001), while the indirect effect through distress is also significant (β = −0.16, *p* < 0.001). This indicates partial mediation, meaning that bullying affects sleep both directly and indirectly by increasing distress. This means that bullying not only harms employees’ sleep on its own but also does so by causing psychological strain. The significant mediation supports the COR proposition that resource loss tends to accumulate, with the initial loss (bullying) leading to further losses (distress) that ultimately impair sleep. Table 6 presents the results of the mediation analysis.

### 4.4. Moderation Analysis

The significant interaction term (PD × RES → SQ, β = 0.18, *p* = 0.003) indicates that Resilience moderates the relationship between Psychological Distress and Sleep Quality, as shown in Table 7. Specifically, the negative impact of psychological distress on sleep quality is weaker among individuals with higher resilience. This suggests that resilient employees can better cope with stress and maintain healthier sleep patterns even under distressing workplace conditions. This moderating effect reinforces COR theory by demonstrating that personal resources—here, resilience—buffer the negative consequences of resource loss, reducing the detrimental impact of distress.

The moderation analysis was further examined using a simple slope test to understand how different levels of Resilience (RES) influence the relationship between Psychological Distress (PD) and Sleep Quality (SQ). The interaction term between PD and RES was significant (β = 0.18, *p* = 0.003), confirming the moderating role of resilience.

The slope analysis shows that resilience significantly moderates the link between psychological distress and sleep quality. When resilience is low (−1 SD), psychological distress has a strong negative impact on sleep quality (β = −0.72), indicating that employees with lower resilience experience poorer sleep under stress. However, when resilience is high (+1 SD), this negative effect weakens (β = −0.36), suggesting that resilient individuals can handle distress more effectively and maintain better sleep. Overall, resilience serves as a protective factor that cushions the impact of stress, supporting the Conservation of Resources (COR) theory by highlighting how resilient people better preserve their mental and physical well-being.

### 4.5. Conditional Moderated Mediation Analysis

Conditional or moderated mediation analysis explores how the indirect effect of one variable on another changes based on the level of a moderating factor. In simpler terms, it combines the ideas of mediation and moderation to explain complex relationships more clearly. This method helps researchers understand not just whether a mediation occurs, but also when and under what conditions it is stronger or weaker. Essentially, the mediator’s influence between the independent and dependent variables is shaped by a third variable that can enhance, reduce, or even reverse the indirect effect [72].

The results in Table 8 highlight a clear and significant moderated mediation effect in which workplace bullying (WPB) influences sleep quality (SQ) through psychological distress (PD), depending on employees’ levels of resilience (RES), supporting Hypothesis H7. When resilience is low (−1 SD), the indirect effect of WPB on SQ through PD is particularly strong and negative (β = −0.44, *p* < 0.001). This means employees with lower resilience are more likely to suffer poor sleep as a result of the psychological distress predicted by workplace bullying. At an average level of resilience, the effect is somewhat weaker (β = −0.33, *p* < 0.001), suggesting that moderate resilience helps cushion the impact. When resilience is high (+1 SD), the negative effect is further reduced (β = −0.23, *p* < 0.001), suggesting that resilience significantly mitigates the harmful link between bullying and sleep problems through distress. The conditional mediation further extends COR theory by showing that the indirect pathway of resource loss is weaker when employees possess higher resilience, highlighting the protective role of personal resources in loss cycles.

The index of moderated mediation (β = 0.11, *p* = 0.006) further confirms that the strength of this indirect relationship varies notably across levels of resilience. The positive and significant value indicates that as resilience grows, the negative influence of workplace bullying on sleep quality, which was channeled through psychological distress, diminishes.

## 5. Discussion

The present study contributes to the growing body of research on workplace bullying by explaining how bullying influences employees’ psychological distress and sleep quality, and by examining the protective role of resilience within this relationship. Grounded in the Conservation of Resources (COR) theory [10], the findings demonstrate that workplace bullying depletes essential emotional resources, thereby disrupting employees’ psychological and physiological well-being. Consistent with COR theory, resilience emerges as a key personal resource that helps buffer against the negative outcomes of bullying-related distress.

To deepen theoretical interpretation, this study’s findings can be further understood through the lens of Conservation of Resources (COR) theory. Workplace bullying acts as a chronic resource-depleting stressor, eroding employees’ emotional and psychological reserves and triggering distress, which subsequently disrupts restorative processes such as sleep. This pattern aligns with prior research demonstrating that persistent interpersonal mistreatment accelerates resource loss cycles and impairs coping capacity. The moderating effect of resilience supports COR’s resource-gain perspective, indicating that individuals with stronger personal resources can interrupt or slow resource-loss spirals. These mechanisms highlight that the bullying–distress–sleep pathway is not merely statistical but reflects a broader dynamic of resource erosion and protection within demanding work environments.

In addition to the Conservation of Resources (COR) perspective, the findings of this study can be interpreted through the lens of the Job Demands–Resources (JD–R) theory. According to JD–R, workplace stress arises when job demands—such as exposure to bullying, high workload, and long working hours—exceed an individual’s available resources, leading to strain and impaired well-being. In the context of IT professionals, bullying represents a significant job demand that depletes emotional and cognitive resources, contributing to psychological distress and poor sleep quality. Conversely, personal resources such as resilience can buffer the negative impact of these demands, enabling employees to cope more effectively and maintain mental health. Integrating JD–R with COR theory provides a complementary explanation: while COR emphasizes the conservation and replenishment of resources, JD–R highlights the dynamic interplay between job demands and personal resources, offering a more comprehensive understanding of the mechanisms linking workplace bullying, distress, and sleep outcomes.

The pattern observed in this study is broadly consistent with prior research conducted internationally. Previous studies by Einarsen and colleagues have consistently demonstrated that exposure to negative acts in the workplace contributes to heightened psychological distress. The present findings mirror this established relationship, suggesting that bullying induces emotional strain regardless of organizational or cultural context [5]. Additionally, earlier work by Niedhammer et al. and Mao et al. [73,74] indicated that chronic workplace stressors are strongly associated with disrupted sleep patterns. The current study aligns with this evidence, showing that psychological strain acts as a meaningful mechanism through which bullying affects employees’ sleep quality.

Furthermore, the results support existing literature that positions psychological distress as a pathway linking workplace mistreatment to physical and psychological outcomes. Similar mediation patterns have been reported in studies examining burnout, anxiety, and somatic complaints, indicating that emotional strain often serves as a bridge between workplace adversity and impaired well-being. This study extends these findings by demonstrating the same mechanism in the IT sector, where intensive workloads, long work hours, and continuous digital engagement may amplify the impact of bullying on rest and recovery [75].

The moderating role of resilience observed in this study also resonates with prior research. Studies by Smith et al. have emphasized that resilience helps individuals withstand stress and maintain healthier psychological functioning under adverse conditions. The present findings reinforce this view by showing that employees with higher resilience experience less deterioration in sleep quality when distressed [76]. However, some studies have reported weaker or non-significant moderation effects, suggesting that the protective influence of resilience may vary across occupational roles, cultural expectations, or the nature of the stressors [77]. By demonstrating a clear buffering effect among Indian IT professionals, this study helps clarify these inconsistencies and highlights the importance of situational factors.

Overall, the findings strengthen the evidence that workplace bullying has both direct and indirect consequences for employee well-being, and that resilience serves as a crucial resource that mitigates these effects. This study not only confirms patterns observed in previous international research but also adds new empirical evidence from the Indian IT sector—a context characterized by high performance demands, long hours, and intense interpersonal interactions. These insights underscore the importance of fostering supportive work environments and enhancing personal coping resources to protect employee health and well-being.

The interpretation of these findings should consider the sample’s demographic characteristics. This study included 380 IT professionals from major metropolitan cities in India, with a reasonably balanced gender mix (57.1% male, 42.9% female). Most participants were young to mid-career professionals, with 54.7% aged 26–35 years and 25.3% aged 36–45, reflecting the demographic backbone of the IT sector. The predominance of younger employees may have influenced the observed levels of psychological distress and the mediating effect on sleep quality, as early-career professionals might have less experience coping with workplace stressors than their older counterparts. Marital status and educational background also provide context. A majority of respondents were married (62.6%), suggesting that many participants might be managing both professional and personal responsibilities, which could exacerbate stress and sleep challenges. Educational qualifications were high, with over half holding a bachelor’s degree and 40.5% a master’s degree, indicating a workforce likely equipped with problem-solving and coping skills, potentially influencing resilience levels. Work experience and job roles further contextualize the findings. Most participants had 1–10 years of experience (72.1%) and were employed in junior or middle management roles (68.2%), while only a minority held senior management positions. This suggests that the findings largely reflect employees navigating early to mid-level career challenges, where exposure to workplace bullying and stress may be more pronounced. Regarding work arrangements, over half (54.5%) were in hybrid setups, with the remainder on-site or fully remote, which could impact the frequency and nature of workplace bullying and, consequently, its effects on psychological distress and sleep quality.

Although the current study did not conduct subgroup analyses, these demographic characteristics offer meaningful interpretive insights. Future research could explore how factors such as age, gender, marital status, job role, and work arrangement moderate the impact of workplace bullying on distress and sleep quality, enabling more tailored interventions to support employee well-being in the IT sector.

This study was conducted among IT professionals in major metropolitan cities in India, a context characterized by hierarchical work structures, collectivist cultural norms, and high organizational loyalty. In such settings, employees may be less likely to openly confront or report bullying behaviors, which can intensify psychological distress. Power distance and respect for authority may influence both the prevalence of workplace bullying and the coping strategies employees adopt. At the same time, collectivist norms and supportive social networks may serve as protective factors, enhancing resilience and buffering the adverse effects of bullying on sleep quality. These cultural characteristics may help explain why resilience emerged as a significant moderator in mitigating the negative impact of psychological distress on sleep among Indian IT professionals. Future research could examine whether these findings hold in less hierarchical or more individualistic cultural contexts to better understand the interplay between culture, workplace bullying, and resilience.

Despite its contributions, this study has several limitations that should be acknowledged. First, the use of a cross-sectional design limits the ability to draw causal inferences about the relationships among workplace bullying, psychological distress, resilience, and sleep quality. Longitudinal or experimental designs would enable stronger conclusions regarding temporal order and causality. Second, this study relies solely on self-report measures, which may introduce response bias and inflate associations due to participants’ subjective perceptions. Future research could incorporate multi-source data, such as supervisor or peer assessments, or objective sleep measures. Third, although procedural remedies were followed, common-method variance cannot be fully ruled out because all variables were collected from the same respondents at a single point in time. Techniques such as time-lagged data collection or marker variables are recommended for future studies. Finally, this study focuses on IT professionals in major Indian metropolitan cities, which may limit generalizability. Expanding future research to other sectors, regions, and cultural contexts could provide a more comprehensive understanding of bullying-related outcomes.

### 5.1. Theoretical Implications

The findings of this study offer several important theoretical contributions to the literature on workplace bullying, psychological distress, resilience, and employee well-being. First, the results reinforce the central assumptions of the Conservation of Resources (COR) theory by demonstrating that workplace bullying functions as a significant threat to employees’ psychological resources. Consistent with earlier research, such as studies by Einarsen et al. and Nielsen [5], the current study shows that exposure to persistent negative acts can deplete emotional resources and heighten vulnerability to strain. This supports the COR perspective that stress arises when individuals face continual resource loss or the threat of such loss [78].

Second, this study strengthens the theoretical understanding of the stressor–strain–outcome pathway by highlighting the mediating role of psychological distress in the relationship between workplace bullying and sleep quality. Prior studies have identified emotional strain as a key mechanism linking interpersonal mistreatment to health outcomes, but research connecting this pathway to sleep quality has been limited. By showing that distress acts as a central psychological process through which bullying affects restorative functions, this study extends existing stress models and reinforces findings from Galanis et al. and others who have suggested that emotional dysregulation can disrupt sleep patterns [78].

Third, the inclusion of resilience as a moderator advances resource-based theories by illustrating how individual differences in resource availability can shape responses to workplace stress. While prior research (e.g., Smith et al. [76], Yu et al. [16]) has conceptualized resilience as a buffering resource, empirical evidence on its role within bullying–distress–sleep models remains scarce. This study provides theoretical support for the COR concept of “resource caravans”, indicating that individuals with stronger personal resources may be better equipped to withstand or recover from the emotional impact of bullying. This adds nuance to existing models by demonstrating that personal strengths can alter the trajectory from stressor to strain and ultimately to health outcomes [79,80].

Finally, by integrating both mediation and moderation into a single conditional process framework, this study contributes to a more holistic theoretical model of employee well-being. It bridges stressor–strain approaches, which focus on emotional reactions to workplace adversity, with resource-based perspectives that emphasize the protective value of personal strengths. This integrated model helps explain how workplace psychological harm progresses to physiological and behavioral consequences, while also highlighting how individual capacities can reshape this progression. Such a comprehensive theoretical perspective strengthens understanding of recovery mechanisms and offers a more complete explanation of how psychosocial stressors impact health and well-being in demanding occupational settings, such as the IT sector.

### 5.2. Practical Implications

The findings of this study offer several practical implications for organizations aiming to improve employee well-being. First, the results demonstrate that workplace bullying is significantly associated with increased psychological distress, which in turn relates to poorer sleep quality. This highlights the need for organizations to implement clear anti-bullying policies and reporting mechanisms that encourage timely identification and resolution of bullying incidents. While the present study does not establish causal relationships, the consistent associations observed suggest that reducing exposure to bullying may help mitigate its negative psychological outcomes.

Second, the moderating role of resilience indicates that employees with higher resilience experienced relatively lower levels of distress in the presence of bullying. Although resilience should not be viewed as a substitute for organizational responsibility, training programs that strengthen coping skills, emotional regulation, and problem-solving may provide an additional layer of protection for employees. Such initiatives should be voluntary and supportive, rather than implying that individuals must independently manage harmful work environments.

Third, organizations may consider integrating stress-management resources—such as counseling services, wellness programs, or Employee Assistance Programs—to support employees experiencing distress. These interventions might help reduce the psychological strain associated with bullying, thereby indirectly improving sleep quality.

Finally, the implications should be interpreted within the limitations of the study design. Given the cross-sectional nature of the data and reliance on self-report measures, these recommendations are presented as potential strategies consistent with the observed relationships rather than definitive causal solutions. Future organizational policies and programs should be informed by longitudinal or intervention-based research.

## 6. Conclusions

This study provides a conceptual understanding of how workplace bullying affects employees’ psychological and physical well-being, particularly sleep quality, through the mediating role of psychological distress and the moderating influence of resilience. Guided by the Conservation of Resources (COR) theory, the findings highlight that bullying functions as a significant stressor that depletes employees’ emotional and mental resources, leading to higher distress levels and poorer sleep. Resilience, however, serves as a personal resource that buffers the negative impact of distress, enabling employees to better maintain mental well-being and recover from workplace stress.

Beyond theoretical contributions, this study underscores several industry-specific considerations. In the IT sector, long working hours, high workload, and remote or hybrid work arrangements may amplify the experience of workplace bullying and its adverse effects on psychological health. These contextual factors, combined with individual differences in resilience, help explain variability in employees’ responses to workplace stressors.

This study also carries practical implications. Organizations should implement policies and interventions that reduce bullying behaviors, promote psychological well-being, and foster resilience among employees. For example, training programs that enhance coping skills, peer-support systems, and resilience-building workshops can help mitigate the negative consequences of workplace stress. Managers should pay particular attention to vulnerable groups, such as less experienced employees or those working remotely, to ensure a supportive work environment.

Finally, this research advances theory by integrating mediation and moderation processes, illustrating how resource loss from workplace bullying can be counterbalanced through resilience. It also contributes to positive organizational psychology by emphasizing the importance of personal resources in maintaining mental health under stress. Overall, this study highlights both the theoretical significance of resilience in workplace stress models and actionable strategies for organizations to enhance employee well-being.

### Limitations and Future Research

While this study offers valuable insights into the interplay between workplace bullying, psychological distress, resilience, and sleep quality, several limitations need to be acknowledged. First, this study relies exclusively on self-reported measures, which may introduce common-method bias and social-desirability effects. Although validated scales were used, the absence of multi-source or objective data limits the robustness of the findings. Second, the cross-sectional design restricts the ability to infer causal relationships among the variables. Longitudinal or experimental designs are recommended to observe how bullying, resilience, and psychological distress evolve over time and to establish clearer temporal sequencing.

Third, although detailed demographic information was collected, no analyses were conducted to examine potential group differences or the role of control variables (e.g., gender, age, job level, or work arrangement). As a result, this study may overlook demographic influences that could shape the relationships among bullying, psychological distress, resilience, and sleep quality. Future research should incorporate such analyses or include relevant control variables to provide a more nuanced understanding of these dynamics.

Fourth, this study’s generalizability is limited, as the sample comprises IT professionals from major metropolitan cities in India. Stressors, interpersonal dynamics, and coping mechanisms may differ substantially across sectors such as healthcare, education, manufacturing, and other service-based industries. Comparative studies across sectors, as well as across cultural contexts, could reveal important contextual variations in how bullying affects sleep and psychological health.

Fifth, the present study focuses primarily on individual-level variables, but workplace bullying is inherently shaped by organizational structures, climates, and leadership styles. Future research should therefore integrate organizational-level factors such as ethical leadership, perceived organizational support, justice perceptions, supervisory behavior, and inclusive HR practices. Examining these factors may clarify how supportive environments buffer the adverse effects of bullying or enhance the protective role of resilience.

Finally, there is a need for intervention-based research that tests practical strategies to strengthen employee well-being. Programs such as resilience-building workshops, mindfulness training, civility interventions, or organizational culture change initiatives could be empirically evaluated to determine their effectiveness in reducing psychological distress and improving sleep quality among employees exposed to workplace bullying.

## Figures and Tables

**Table 1:** Reliability and Validity.

Construct	No. of Items	Avg. Factor Loading	Range of Factor Loadings	CA	CR	AVE
Workplace Bullying (WPB)	21	0.72	0.62–0.83	0.94	0.95	0.55
Psychological Distress (PD)	10	0.76	0.65–0.84	0.91	0.93	0.58
Sleep Quality (SQ)	18	0.69	0.58–0.79	0.88	0.90	0.52
Resilience (RES)	25	0.73	0.61–0.84	0.96	0.97	0.57

Note: CA—Cronbach’s Alpha; CR—Composite Reliability; AVE—The Average Variance Extracted.

**Table 2:** Discriminant Validity.

Constructs	WPB	PD	SQ	RES
Workplace Bullying (WPB)	—			
Psychological Distress (PD)	0.74	—		
Sleep Quality (SQ)	0.68	0.71	—	
Resilience (RES)	0.42	0.56	0.49	—

**Table 3:** VIF Values.

Endogenous Construct	Predictor Constructs	VIF Value
Psychological Distress (PD)	Workplace Bullying (WPB)	1.87
Sleep Quality (SQ)	Workplace Bullying (WPB)	1.64
	Psychological Distress (PD)	2.05
	Resilience (RES)	1.58

**Table 4:** R^2^ Value and f^2^ Value.

Endogenous Construct	Predictor Construct(s)	R^2^ Value	f^2^ (Effect Size)	Interpretation of f^2^
Psychological Distress (PD)	Workplace Bullying (WPB)	0.56	0.32	Large
Sleep Quality (SQ)	Workplace Bullying (WPB)		0.14	Medium
	Psychological Distress (PD)	0.48	0.28	Large
	Resilience (RES)		0.10	Small to Medium

**Table 5:** Path Coefficient.

Path Relationship	Coefficient (β)	Standard Deviation (SD)	T Value	*p* Value	Result
WPB → SQ	−0.32	0.06	5.33	<0.001	H1 Supported
WPB → PD	0.61	0.07	8.71	<0.001	H2 Supported
PD → SQ	−0.54	0.08	6.75	<0.001	H3 Supported
RES → SQ	0.28	0.07	4.00	<0.001	H5 Supported

**Table 6:** Mediation Analysis.

Relationship (Path)	Type of Effect	Coefficient (β)	Standard Deviation (SD)	T Value	*p* Value	Result
WPB → SQ (without mediator)	Total Effect	−0.48	0.09	5.33	<0.001	H4 Supported
WPB → SQ	Direct Effect	−0.32	0.06	5.33	<0.001	
WPB → PD → SQ	Indirect Effect (Mediation)	−0.16	0.06	2.67	<0.001	

**Table 7:** Moderation Analysis.

Relationship (Path)	Coefficient (β)	Standard Deviation (SD)	T Value	*p* Value	Result
PD × RES → SQ	0.18	0.06	3.00	<0.001	H6 Supported

**Table 8:** Conditional Moderated Mediation Analysis.

Conditional Effect of WPB on SQ via PD	Level of Resilience	Standard Deviation (SD)	T Value	*p* Value
Low Resilience (−1 SD)	β = −0.44	0.07	6.29	<0.001
Mean Resilience (Mean)	β = −0.33	0.06	5.50	<0.001
High Resilience (+1 SD)	β = −0.23	0.05	4.60	<0.001
Index of Moderated Mediation	β = 0.11	0.04	2.75	<0.001

## Data Availability

The original contributions presented in this study are included in the article material. Further inquiries can be directed to the corresponding author.

## Appendix A. Workplace Bullying Scale

**Table A1:** Workplace Bullying Scale.

S. No.	Statements	5	4	3	2	1
1	Shifting work tasks without your consultation					
2	Undervaluing of your work					
3	Being ordered to do work below your level of proficiency					
4	Persistent unjustified monitoring of your work					
5	Repeated attempts to undermine your personal dignity					
6	Verbal and non-verbal threats					
7	Making inappropriate jokes about you					
8	Withholding necessary information affecting your professional progress					
9	Exclude you from workgroup activities					
10	Reject your application for leave, training or promotion without reason					
11	Setting of impossible deadlines to accomplish work					
12	Spread rumours about you					
13	Repeated offensive remarks about your person or private life					
14	Signals from others that you should resign your job					
15	Repeated reminders of your mistakes					
16	Neglect of your opinions or views					
17	Not give importance to your rights and opinions with reference to your gender					
18	Devaluation of your rights and opinions with reference to your age					
19	Negative responses from others because you work hard					
20	Several times forced to attend supplementary meetings and training sessions					
21	Intimidatory use of discipline/competence procedure					

Note: five-point Likert scale ranging from 1 (Strongly Disagree) to 5 (Strongly Agree).

## Appendix B. Psychological Distress Scale

**Table A2:** Psychological Distress Scale.

S. No.	Statements	5	4	3	2	1
1	During the last 4 weeks, about how often did you feel tired out for no good reason?					
2	During the last 4 weeks, about how often did you feel nervous?					
3	During the last 4 weeks, about how often did you feel so nervous that nothing could calm you down?					
4	During the last 4 weeks, about how often did you feel hopeless?					
5	During the last 4 weeks, about how often did you feel restless or fidgety?					
6	During the last 4 weeks, about how often did you feel so restless you could not sit still?					
7	During the last 4 weeks, about how often did you feel depressed?					
8	During the last 4 weeks, about how often did you feel that everything was an effort?					
9	During the last 4 weeks, about how often did you feel so sad that nothing could cheer you up?					
10	During the last 4 weeks, about how often did you feel worthless?					

Note: five-point Likert scale ranging from 1 (Strongly Disagree) to 5 (Strongly Agree).

## Appendix C. Sleep Quality Scale

**Table A3:** Sleep Quality Scale.

S. No.	Statements	5	4	3	2	1
1	I have difficulty falling asleep.					
2	I fall into a deep sleep.					
3	I wake up while sleeping.					
4	I have difficulty getting back to sleep once I wake up in the middle of the night.					
5	I wake up easily because of noise.					
6	I toss and turn.					
7	I never go back to sleep after awakening during sleep.					
8	I feel refreshed after sleep.					
9	I feel unlikely to sleep after sleep.					
10	Poor sleep gives me headaches.					
11	Poor sleep makes me irritated.					
12	I would like to sleep more after waking up.					
13	My sleep hours are enough.					
14	Poor sleep makes me lose my appetite.					
15	Poor sleep makes hard for me to think.					
16	I feel vigorous after sleep.					
17	Poor sleep makes me lose interest in work or others.					
18	My fatigue is relieved after sleep.					
19	Poor sleep causes me to make mistakes at work.					
20	I am satisfied with my sleep.					
21	Poor sleep makes me forget things more easily.					
22	Poor sleep makes it hard to concentrate at work.					
23	Sleepiness interferes with my daily life.					
24	Poor sleep makes me lose desire in all things.					
25	I have difficulty getting out of bed.					
26	Poor sleep makes me easily tired at work.					
27	I have a clear head after sleep.					
28	Poor sleep makes my life painful.					

Note: five-point Likert scale ranging from 1 (Strongly Disagree) to 5 (Strongly Agree).

## Appendix D. Resilience Scale

**Table A4:** Resilience Scale.

S. No.	Statements	5	4	3	2	1
1	When I make plans I follow through with them.					
2	I usually manage one way or another.					
3	I am able to depend on myself more than anyone else.					
4	Keeping interested in things is important to me.					
5	I can be on my own if I have to.					
6	I feel proud that I have accomplished things in life.					
7	I usually take things in my stride.					
8	I am friends with myself.					
9	I feel that I can handle many things at a time.					
10	I am determined.					
11	I seldom wonder what the point of it all is.					
12	I take things one day at a time.					
13	I can get through difficult times because I’ve experienced difficulty before.					
14	I have self-discipline.					
15	I keep interested in things.					
16	I can usually find something to laugh about.					
17	My belief in myself gets me through hard times.					
18	In an emergency, I’m somebody people generally can rely on.					
19	I can usually look at a situation in a number of ways.					
20	Sometimes I make myself do things whether I want to or not.					
21	My life has meaning.					
22	I do not dwell on things that I can’t do anything about.					
23	When I am in a difficult situation, I can usually find my way out of it.					
24	I have enough energy to do what I have to do.					
25	It’s okay if there are people who don’t like me.					

Note: five-point Likert scale ranging from 1 (Strongly Disagree) to 5 (Strongly Agree).

## Appendix E. Demographic Questionnaire

Demographic Information

Please select the most appropriate response for each item. All information will be used for research purposes only and will remain confidential.

1. Gender
  - ☐ Male
  - ☐ Female
  - ☐ Prefer not to say
2. Age Group
  - ☐ 18–25 years
  - ☐ 26–35 years
  - ☐ 36–45 years
  - ☐ Above 45 years
3. Marital Status
  - ☐ Single
  - ☐ Married
  - ☐ Other
4. Highest Educational Qualification
  - ☐ Diploma/Professional Certification
  - ☐ Bachelor’s Degree
  - ☐ Master’s Degree
  - ☐ Other (please specify): _____________
5. Total Work Experience
  - ☐ Less than 1 year
  - ☐ 1–5 years
  - ☐ 6–10 years
  - ☐ 11–15 years
  - ☐ More than 15 years
6. Current Job Role/Level
  - ☐ Entry-Level/Associate
  - ☐ Junior/Middle Management
  - ☐ Senior Management
  - ☐ Other (please specify): _____________
7. Work Arrangement
  - ☐ On-site
  - ☐ Remote
  - ☐ Hybrid
8. Location/City of Employment
  - ☐ Bengaluru
  - ☐ Chennai
  - ☐ Hyderabad
  - ☐ Mumbai
  - ☐ Pune
